# Supplementary material for: Optimal minimal residual disease threshold in pediatric acute myeloid leukemia: A retrospective cohort study based on the TARGET database
Source: PLoS Med. 2026 May 8;23(5):e1005088. doi: 10.1371/journal.pmed.1005088 (PMC13155632; doi:10.1371/journal.pmed.1005088)
Supplement: S1 Code — (ZIP) [file pmed.1005088.s002.zip › S2 code/PROJ8_1_tbl1/PROJ8_1_tbl1.htm]

## Ô¤²âÄ£ÐÍÓëROC·ÖÎö

Please use EmpowerStats ver. 2.2 or 3.0 since R packages updated, results from this version may be inaccurate.
Outcome:
First Event
Time:
X28

Model 0: Multiple Fractional Polynomial model from observed data
-0.00007\*I(((X23 + 0.1)/10)^-2) +0.32927\*I(((X23 + 0.1)/10)^1)

|  |  |  |  |  |  |  |
| --- | --- | --- | --- | --- | --- | --- |
|  | coeff. | se. | HR | Low 95%CI | High 95%CI | P value |
| I(((X23 + 0.1)/10)^-2) | -0.0001 | 0.0000 | 0.9999 | 0.9999 | 0.9999 | <0.0001 |
| I(((X23 + 0.1)/10)^1) | 0.3293 | 0.0314 | 1.3900 | 1.3069 | 1.4782 | <0.0001 |

rsq = 0.1631, maxrsq = 0.9989
, Log likelihood -4014.3440

|  |  |  |  |  |  |  |
| --- | --- | --- | --- | --- | --- | --- |
| concordant | discordant | tied.risk | tied.time | std(c-d) | C | se(C) |
| 208863 | 78630 | 249643 | 1231 | 9225.442 | 0.6212 | 0.0086 |

Create new variables for Nomogram: 
V.new1 = I(((X23+0.1)/10)^-2)V.new2 = I(((X23+0.1)/10)^1)
Model 1: Full model from observed data
0.04433\*X23

|  |  |  |  |  |  |  |
| --- | --- | --- | --- | --- | --- | --- |
|  | coeff. | se. | HR | Low 95%CI | High 95%CI | P value |
| X23 | 0.0443 | 0.0026 | 1.0453 | 1.0400 | 1.0506 | <0.0001 |

rsq = 0.1277, maxrsq = 0.9989
, Log likelihood -4039.3423

|  |  |  |  |  |  |  |
| --- | --- | --- | --- | --- | --- | --- |
| concordant | discordant | tied.risk | tied.time | std(c-d) | C | se(C) |
| 208863 | 78630 | 249643 | 1231 | 9225.442 | 0.6212 | 0.0086 |

|  |  |  |  |  |  |  |  |  |  |
| --- | --- | --- | --- | --- | --- | --- | --- | --- | --- |
| Obs | Events | Model L.R. | d.f. | P.value | Score | Score P | R2 | g | gr |
| 1205 | 611 | 164.59 | 1 | <0.0001 | 434.0289 | 0 | 0.1278 | 0.2576 | 1.2938 |

Ô¤²âÄ£ÐÍROCÇúÏß·ÖÎö¼°×î¼ÑãÐÖµ·ÖÎö

|  |  |  |  |  |  |  |  |  |  |  |
| --- | --- | --- | --- | --- | --- | --- | --- | --- | --- | --- |
| Model | Time | Best.cut.X | Best.cut.sens | Best.cut.spec | Cases | N.survivor | N.censored | Cum.incidence | Surv.prob | AUC |
| mfp | 0.38 | 2.70259575983228 | 0.68 | 0.817 | 122 | 1077 | 6 | 0.101 | 0.897 | 0.775 |
| mfp | 0.56 | 2.83430525710882 | 0.571 | 0.816 | 182 | 1012 | 11 | 0.151 | 0.8428 | 0.711 |
| mfp | 0.7 | 2.83430525710882 | 0.483 | 0.821 | 242 | 952 | 11 | 0.201 | 0.7929 | 0.669 |
| mfp | 0.81 | 2.83430525710882 | 0.452 | 0.832 | 305 | 887 | 13 | 0.254 | 0.7395 | 0.656 |
| mfp | 0.92 | 3.26236096393524 | 0.468 | 0.821 | 363 | 831 | 11 | 0.302 | 0.6937 | 0.655 |
| mfp | 1.09 | 3.26236096393524 | 0.436 | 0.827 | 426 | 767 | 12 | 0.355 | 0.6435 | 0.641 |
| mfp | 1.38 | 3.26236096393524 | 0.425 | 0.84 | 487 | 694 | 24 | 0.406 | 0.5902 | 0.64 |
| Full | 0.38 | 2.87669790263658 | 0.68 | 0.817 | 122 | 1077 | 6 | 0.101 | 0.897 | 0.775 |
| Full | 0.56 | 3.05402782669744 | 0.571 | 0.816 | 182 | 1012 | 11 | 0.151 | 0.8428 | 0.711 |
| Full | 0.7 | 3.05402782669744 | 0.483 | 0.821 | 242 | 952 | 11 | 0.201 | 0.7929 | 0.669 |
| Full | 0.81 | 3.05402782669744 | 0.452 | 0.832 | 305 | 887 | 13 | 0.254 | 0.7395 | 0.656 |
| Full | 0.92 | 3.63035007989523 | 0.468 | 0.821 | 363 | 831 | 11 | 0.302 | 0.6937 | 0.655 |
| Full | 1.09 | 3.63035007989523 | 0.436 | 0.827 | 426 | 767 | 12 | 0.355 | 0.6435 | 0.641 |
| Full | 1.38 | 3.63035007989523 | 0.425 | 0.84 | 487 | 694 | 24 | 0.406 | 0.5902 | 0.64 |

×î¼ÑãÐÖµÈ¡Ãô¸Ð¶È+ÌØÒì¶È×î´óµÄ·Ö½çÖµ¡£¸÷·Ö½çµã¶ÔÓ¦µÄÃô¸Ð¶ÈÌØÒì±£´æÔÚROCÊä³öÎÄ¼þ£¨.xls£©Àï
Inverse Probability of Censoring Weighting (IPCW) estimates of Cumulative/Dynamic time-dependent ROC curve.
